# Natural cycle versus modified natural cycle for endometrial preparation in women undergoing frozen-thawed embryo transfer: An RCT

**DOI:** 10.18502/ijrm.v20i11.12359

**Published:** 2022-12-10

**Authors:** Maryam Farid Mojtahedi, Saeedeh Aref, Ashraf Moini, Arezoo Maleki-Hajiagha, Ladan Kashani

**Affiliations:** ^1^Endocrinology and Female Infertility Unit, Arash Women's Hospital, Tehran University of Medical Sciences, Tehran, Iran.; ^2^Department of Endocrinology and Female Infertility, Reproductive Biomedicine Research Center, Royan Institute for Reproductive Biomedicine, ACECR, Tehran, Iran.; ^3^Department of Anatomy, School of Medicine, Tehran University of Medical Sciences, Tehran, Iran.; ^4^Research Development Center, Arash Women's Hospital, Tehran University of Medical Sciences, Tehran, Iran.

**Keywords:** Embryo transfer, In vitro fertilization, Pregnancy rate, Live birth, Human chorionic gonadotropin.

## Abstract

**Background:**

Studies have evaluated different endometrial preparation methods, but the optimal frozen-thawed embryo transfer (FET) cycle strategy in terms of the in-vitro fertilization outcome is still debated.

**Objective:**

To compare the natural versus modified natural cycles for endometrial preparation in women undergoing FET.

**Materials and Methods:**

This study was designed as a randomized clinical trial, and it was performed at the Arash women's hospital between August 2016-2018. Hundred and forty eligible participants were enrolled in this study and were randomly divided into 2 groups by using the block randomization method, including true natural FET (n = 70) and modified natural FET (mNFET) (n = 70) cycles. Both groups were monitored for endometrial thickness and follicular size; simultaneously spontaneous luteinizing hormone surge using urinary luteinizing hormone testing kits. The mNFET group received 5000 IU of human chorionic gonadotropin injection to trigger final follicular maturation. Luteal support by vaginal progesterone (cyclogest 400 mg twice daily) was used in true natural FET from the day of transfer until the 10 ${}^{\text{th}}$ wk of pregnancy. Chemical and clinical pregnancy and abortion rates were considered as the primary outcomes.

**Results:**

There were no differences in the participants' baseline characteristics between groups. There was no difference in clinical pregnancy and abortion rate between groups, while the implantation rate was significantly higher in the mNFET group (29.2% vs. 17.6%; p = 0.036).

**Conclusion:**

The results demonstrated that both types of natural cycles were similar in pregnancy outcomes, while modified cycles might be associated with a higher implantation rate.

## 1. Introduction

One of the most challenging issues in frozen-thawed embryo transfer (FET) cycles is a selection of an appropriate endometrial preparation method that provides the most endometrial receptivity and synchronization between embryonic and endometrial development. There are 3 main cycle regimens for endometrial preparation: natural cycle, ovulation induction cycle, and artificial cycle. The natural cycle can only be used in normoovulatory women. Several studies have evaluated the advantages and disadvantages of each method, but the optimal FET cycle strategy in terms of the in-vitro fertilization outcomes is still debated (1). There is no need for hormone therapy in the natural cycle. Endometrial preparation is performed under physiological conditions; however, there are concerns about adverse maternal and fetal outcomes in pregnancies undergoing artificial cycles (2-7).

Depending on whether ovulation is spontaneous or induced, there are 2 different types of natural cycles: the true natural FET (tNFET) cycle with spontaneous luteinizing hormone (LH) surge and the modified natural FET (mNFET) cycle, which needs the administration of a bolus of human chorionic gonadotropin (hCG). Detection of ovulation in the tNFET cycle requires numerous monitoring visits. While using hCG in the mNFET cycle provides more flexibility and facilitates FET planning, scheduling ET in the natural cycle is a little difficult. Moreover, the number of visits in the mNFET group is significantly lower compared to the tNFET; therefore, it is more cost-effective and convenient for patients (8).

Few studies have compared these 2 regimens with conflicting results. Since there is still insufficient evidence to support the use of either of these regimens and there are few adequately powered randomized clinical trials (RCTs), this RCT was conducted to compare ET outcomes of the tNFET versus the mNFET cycle.

## 2. Materials and Methods

This randomized, controlled trial was performed between August 2016 and August 2018. The inclusion criteria were women aged < 38 yr old with a normal body mass index (BMI) (19.8-24.9), having frozen embryos, regular menstrual cycles (25-35 days), and a history of < 3 previous ET cycles. The exclusion criteria were uterine malformations, severe endometriosis (stage 3 or more according to American Fertility Society), polycystic ovary syndrome, follicle-stimulating hormone (FSH) more than 12 IU/ml, and severe male factor infertility.

The participants underwent FET according to a computer-generated concealed randomization list using the block randomization method. There were 6 blocks. The statistician prepared random treatment assignments in sealed envelopes to conceal the randomization list from all research staff involved in enrollment and assessment.

According to the randomization list, 50 participants underwent the mNFET cycle, and the other parallel group underwent the tNFET cycle. In this study, the vitrification method was used for the cryopreservation of embryos. The outcome investigators (infertility clinic's midwife and the gynecologist who performed ultrasound assessments) and the statistician were unaware of the type of intervention, until the end of the study.

In the tNFET cycle group, spontaneous LH surge was monitored using urinary LH testing kits. The participants were instructed to use urinary LH kits daily from the 10 ${}^{\text{th}}$ day of the menstrual cycle. Four days after a positive LH test, thawed embryos were transferred. In the mNFET group, follicular growth was monitored using regular ultrasound assessments. Simultaneously, urinary LH kits were used daily from the 10 ${}^{\text{th}}$ day of the menstrual cycle. In the presence of an endometrial thickness of ≥ 7 mm and a dominant follicle with a diameter of ≥ 17 mm, 5000 IU hCG was injected to trigger ovulation if a positive LH test was not detected. The embryos were then transferred after 5 days.

The luteal phase was supported by a vaginal progesterone suppository at a dose of 400 mg twice daily in the tNFET group. In the mNFET group, hCG was assumed enough for endometrial preparation and luteal phase support (LPS). Depending on the participant's age, embryo quality, and hormonal status, 1-3 high-quality embryos (A and AB) were transferred by ultrasound guide 4 days after spontaneous LH rise and 5 days after the hCG trigger. All embryos in both groups were on the third day of the developmental stage. The embryos were thawed in the early morning of the transfer day. Survival of the embryos was morphologically evaluated once warming was completed. Each embryo was rated on a scale of A (excellent) to D (poor) according to the Gardner scaling system (9).

In the present study, the rates of clinical pregnancy, chemical pregnancy, and abortion were considered as the primary outcomes. The implantation, ongoing pregnancy rates, number of embryos transferred, and endometrial thickness before ET were the secondary outcomes of this study. The endometrial thickness was evaluated by transvaginal ultrasound, and the number of transferred embryos was extracted from the embryologist's report.

Two wk after ET, serum β-hCG level was measured. A positive β-hCG test was considered as a positive chemical pregnancy. In participants with a chemical pregnancy, an ultrasound assessment was performed to evaluate the presence of the gestational sac and the fetal heartbeat to establish the clinical pregnancy. For calculation of the implantation rate, the number of gestational sacs observed in ultrasound were divided by the number of transferred embryos. Also, we considered pregnancies that were continued more than 12 wk, as ongoing pregnancies. A spontaneous pregnancy loss before completion of 20 ${}^{\text{th}}$ wk, were defined as miscarriage and accordingly the abortion rate was calculated by dividing the total number of miscarriages by the total number of pregnancies.

### Ethical considerations

This research was approved by the Ethics Committee of Tehran University of Medical Sciences, Tehran, Iran (Code: IR.TUMS.MEDICINE.REC.1395.426). The trial was performed according to the Declaration of Helsinki and subsequent revisions. All participants signed informed consent before entering the study. In addition, the final approved study protocol is available at the Iranian Registry of Clinical Trials (Date of last update: 2021-12-01).

### Statistical analysis

The continuous variables were analyzed by student's *t *test and categorical variables were analyzed by Chi-square or Fisher's exact test (were each were applicable). P-value ≤ 0.05 were considered statistically significant. We used the SPSS software (version 16.0, Chicago, Illinois, USA) for statistical analyses.

## 3. Results

**Figure 1:**
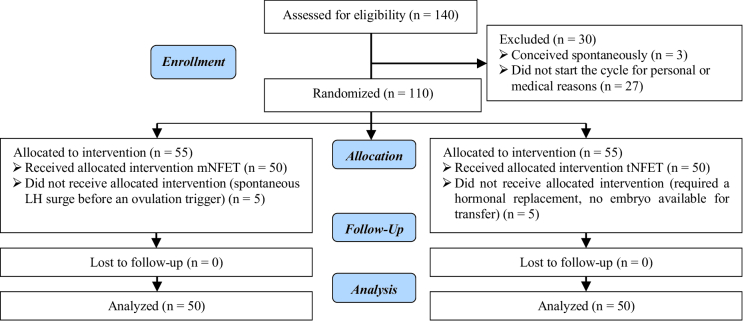
Flow diagram of study.

Hundred and forty participants were enrolled in this study. Three women became spontaneously pregnant before starting the FET cycle, and 27 did not underwent FET cycle because of personal or medical issues. Finally, 110 women started the FET cycle. In the tNFET group, 2 participants required hormonal replacement therapy due to the absence of follicular development and 3 participants had no embryos available for transfer after thawing. In the mNFET group, 5 participants had a spontaneous LH surge before the ovulation trigger. So, 5 participants from each group were excluded from the study, and 50 participants in each group were included in the final analysis. More details about participants' enrollment are provided in the study flow diagram (Figure 1).

No significant differences were observed in the demographics or clinical characteristics between the 2 groups. No differences were observed in the baseline hormonal level. Cycle characteristics, including the number of oocytes and embryos, were similar (Table I).

Endometrial thickness was similar in the tNFET and mN-FET groups. There were no differences in the chemical pregnancy, clinical pregnancy, and abortion rate between the 2 groups; however, the implantation rate was significantly higher in the mN-FET group than the tNFET group (29.2% vs. 17.6%; p = 0.036) (Table II).

**Table 1:** Demographic characteristics of participants in study groups


**Characteristics**		**tNFET group (n = 50)**	**mNFET group (n = 50)**	**P-value**
Participants age (yr)*		31.7 ± 4.8	32.5 ± 3.5	0.36
Male age (husbands) (yr)*		36.3 ± 5.2	35.8 ± 4.0	0.58
BMI (kg/m^2^)*		22.80 ± 2.24	22.86 ± 1.30	0.87
AMH (ng/mL)*		2.37 ± 1.86	2.62 ± 1.27	0.43
Basal FSH (mIU/ml)*		6.2 ± 1.7	6.5 ± 1.5	0.34
Basal LH (mIU/ml)*		5.8 ± 2.5	5.5 ± 2.3	0.62
No. of previous IVF attempts* ${}^{†}$		0.6 ± 0.79	0.4 ± 0.67	0.06
No. oocytes retrieved in fresh cycle*		13.9 ± 5.3	13.1 ± 6.2	0.48
Type of infertility***				
	**Primary**	44 (88)	41 (82)	
	**Secondary**	6 (12)	9 (18)	0.28
Causes of infertility**				
	**Tubal**	2 (4)	1 (2)	
	**Endometriosis**	1 (2)	3 (6)	
	**Male factor**	37 (74)	31 (62)	0.21
	**Unexplained**	9 (18)	11 (22)	
	**Tubal and male factor**	1 (2)	4 (8)	
*Data presented as Means ± standard deviation. Student's *t* test. **Data presented as number (%). Fisher's exact test. ***Data presented as number (%). Chi-square test. ${}^{†}$ The interquartile range = 1, tNFET: True natural frozen embryo transfer, mNFET: Modified natural frozen embryo transfer, BMI: Body mass index, AMH: Anti-Müllerian hormone, FSH: Follicle-stimulating hormone, LH: Luteinizing hormone, IVF: In vitro fertilization				

**Table 2:** Comparison of study outcomes between groups


**Characteristic/outcome**	**tNFET group**	**mNFET group**	**P-value**
Chemical pregnancy*	28 (56)	21 (42)	0.16
Clinical pregnancy*	18 (36)	27 (54)	0.07
Implantation rate*	21 (17.6)	35 (29.2)	0.03
Ongoing pregnancy*	17 (34)	24 (48)	0.15
Abortion*	1 (5.6)	3 (11.1)	0.52
No. of embryos transferred**	2.3 ± 0.4	2.2 ± 0.6	0.10
Endometrial thickness (mm)**	9.45 ± 1.26	9.57 ± 1.13	0.64
*Data presented as n (%). Chi-square test. **Data presented as Means ± standard deviation. Student's *t* test, tNFET: True natural frozen embryo transfer, mNFET: Modified natural frozen embryo transfer			

## 4. Discussion

This study was conducted to compare 2 methods of endometrial preparation: tNFET and mNFET cycle. No significant differences were found in the pregnancy outcomes, while the implantation rate was significantly higher in the mNFET group.

A crucial factor for implantation success is synchronization between the embryo and the endometrial developmental stage. The implantation window, when the endometrium has the highest receptivity for the embryo, is regulated by ovulation triggering signals and, most importantly, by the LH surge. As a result, the cornerstone of the success of both natural cycles is based on the accurate detection of the LH surge and consequently identification of the exact time of ovulation. In the mNFET cycle, the LH surge is controlled by hCG injection, so the exact time of ovulation is almost determined. In tNFET cycles, however, due to difficulties in accurate detection of the ovulation time, cycle cancellation occurs in 6% of the participants (2). Additionally, hCG may induce ovulation and initiate the same cascade of events leading to a receptive endometrium in a flexible scheduled program (10, 11).

Along with the difficulties of using the tNFET method, it should be noted that it is the method of choice for normal ovulatory women due to its ease of use, fewer side effects, and lower medical costs (11). However, this method needs several cycle monitoring for the programming of ET according to the time of ovulation (12). On the other hand, although the use of hCG lowers the clinical workload, its role in improving the clinical outcomes of the FET cycles remains limited.

The interventional design of this study was one of its strengths as most of previous studies investigated the difference between these 2 methods using an observational design. To date, few RCTs and systematic reviews have compared these 2 regimens with each other or with other regimens, but there were no conclusive results (1, 3, 13-16). A recent large study showed a higher clinical pregnancy and live birth rates following FET in hCG-triggered ovulation cycles compared to NCFET and artificial cycle-FET (17).

A study reported that hCG had a negative impact on the endometrium and reduced the ongoing pregnancy rate compared to spontaneous natural cycle. However, in this study, luteal support was administered neither in the hCG nor in the natural cycle group (10). Another study criticized this paper for the timing of the embryo thaw and transfer and for not using vaginal progesterone for luteal support as the factors that could have affected the outcome. As they mentioned, hCG worked beautifully; it simplified the monitoring process without compromising the live birth rate (18). Our decision to transfer embryos 4 days after a spontaneous LH rise and 5 days after the hCG trigger was based on a theory that ovulation might occur at a later stage after hCG administration compared to the spontaneous LH surge (3). It has been already established that HCG administration after endogenous LH rise negatively influences the pregnancy rate in mNFET (19). We used urinary LH measurement instead of serum hormone monitoring for participants' convenience. Although we were lucky enough to have positive test results for all NC-FET participants, this method has some drawbacks that need to be addressed. False-negative result of urine LH might occur as a result of diluted urine and LH surges of short duration or low peak values (14).

Results of studies that evaluated the benefits of LPS in mNFET are inclusive and heterogeneous (11, 15, 20, 21). It seems that early initiation of progesterone on the day of hCG or following the LH rise in tNFET may have a detrimental effect on endometrial development and result in the early closure of the implantation window. A higher live birth rate was demonstrated by initiating progesterone on the evening of the day of ET (11, 22). Mackens and co-workers performed both tNFET and mNFET without LPS and found no significant differences in CPR, which confirms the hypothesis that improper timing of LPS may affected results of the previous retrospective studies (14). Some recent studies suggested that the boost of hCG injection given to participants in tNFETs resulted in improved cycle outcomes (20, 23, 24). It has been demonstrated that natural cycle is superior to spontaneous ovulatory cycle, as less visits are required because of ovulation triggering by hCG injection, and it is not associated with detrimental effect on implantation, clinical pregnancy, live birth, and abortion rates (25). We used hCG in mNFET for triggering and luteal support.

In our study, progesterone was used as LPS in the natural cycle group starting 2 days after spontaneous LH surge. Although it is not contrary to current opinions, its initiation on the transfer day might have been better.

In a very recent study, transfer was not programmed according to the LH surge, nor was hCG used for ovulation trigger. Their main concept was that treatment success depends on proper ET timing in relation to the progesterone rising, whether endogenous or exogenous. This new concept is a physician-friendly strategy if its success is confirmed in future trials (26).

One of the main limitations of the present study was its small sample size. A non-significant but remarkable difference was found in the clinical pregnancy rate (54% in the mNFET group vs. 36% in the tNFET group). In addition, the mNFET group showed a significantly higher implantation rate than the tNFET group (29.2% vs. 17.6%). However, although such a difference exists, it is not statistically significant enough to draw a definite conclusion. Therefore, considering the probable clinical importance of this issue, the results should be interpreted with caution, and more RCTs with larger sample sizes are still required. Furthermore, it is better to administer progesterone for endometrial development on the day of transfer than after LH surge. It might negatively affect implantation in the tNFET group, which warrants more investigation.

## 5. Conclusion

Both types of natural cycles were similar in pregnancy outcomes, while the mNFET may be associated with a higher implantation rate. Thanks to the fewer visits needed in the mNFET group and similar pregnancy outcomes, the mNFET cycle may be more convenient and preferable. However, considering the small sample size of the present study, more extensive clinical trials are needed for a definite conclusion.

##  Conflict of Interest

The authors declare that there is no conflict of interest.

## References

[B1] Ghobara T, Gelbaya TA, Ayeleke RO (2017). Cycle regimens for frozen‐thawed embryo transfer. Cochrane Database Syst Rev.

[B2] Mubarak S, Acharyya S, Viardot-Foucault V, Tan H, Phoon J (2019). A comparison of the miscarriage and live birth rate for frozen embryo transfer according to two endometrial preparations: Natural or primed with estrogens. Fertil Reprod.

[B3] Mackens S, Santos-Ribeiro S, Van De Vijver A, Racca A, Van Landuyt L, Tournaye H, et al (2017). Frozen embryo transfer: A review on the optimal endometrial preparation and timing. Hum Reprod.

[B4] Wang B, Zhang J, Zhu Q, Yang X, Wang Y (2020). Effects of different cycle regimens for frozen embryo transfer on perinatal outcomes of singletons. Hum Reprod.

[B5] Dall’Agnol H, Velasco JAG

[B6] Zong L, Liu P, Zhou L, Wei D, Ding L, Qin Y (2020). Increased risk of maternal and neonatal complications in hormone replacement therapy cycles in frozen embryo transfer. Reprod Biol Endocrinol.

[B7] Ginstrom Ernstad E, Wennerholm UB, Khatibi A, Petzold M, Bergh C (2019). Neonatal and maternal outcome after frozen embryo transfer: Increased risks in programmed cycles. Am J Obstet Gynecol.

[B8] Isikoglu M, Aydinuraz B, Avci A, Ceviren AK (2020). Modified natural protocol seems superior to natural and artificial protocols for preparing the endometrium in frozen embryo transfer cycles. Minerva Ginecol.

[B9] Sakkas D, Gardner DK (2018). Evaluation of embryo quality analysis of morphology and physiology.

[B10] Mousavi Fatemi H, Kyrou D, Bourgain C, Van den Abbeel E, Griesinger G, Devroey P (2010). Cryopreserved-thawed human embryo transfer: Spontaneous natural cycle is superior to human chorionic gonadotropin-induced natural cycle. Fertil Steril.

[B11] Mumusoglu S, Polat M, Ozbek IY, Bozdag G, Papanikolaou EG, Esteves SC, et al (2021). Preparation of the endometrium for frozen embryo transfer: A systematic review. Front Endocrinol.

[B12] Wu H, Zhou P, Lin X, Wang S, Zhang S (2021). Endometrial preparation for frozen-thawed embryo transfer cycles: A systematic review and network meta-analysis. J Assist Reprod Genet.

[B13] Huberlant S, Vaast M, Anahory T, Tailland ML, Rougier N, Ranisavljevic N, et al (2018). [Natural cycle for frozen-thawed embryo transfer: Spontaneous ovulation or triggering by HCG]. Gynecol Obstet Fertil Senol.

[B14] Mackens S, Stubbe A, Santos-Ribeiro S, Van Landuyt L, Racca A, Roelens C, et al (2020). To trigger or not to trigger ovulation in a natural cycle for frozen embryo transfer: A randomized controlled trial. Hum Reprod.

[B15] Huber WJ, Sauerbrun‐Cutler MT, Krueger PM, Sharma S (2021). Novel predictive and therapeutic options for better pregnancy outcome in frozen embryo transfer cycles. Am J Reprod Immunol.

[B16] Madani T, Ramezanali F, Yahyaei A, Hasani F, Bagheri Lankarani N, Mohammadi Yeganeh L (2019). Live birth rates after different endometrial preparation methods in frozen cleavage-stage embryo transfer cycles: A randomized controlled trial. Arch Gynecol Obstet.

[B17] Levi Setti PE, Cirillo F, De Cesare R, Morenghi E, Canevisio V, Ronchetti C, et al (2020). Seven years of vitrified blastocyst transfers: Comparison of 3 preparation protocols at a single ART center. Front Endocrinol.

[B18] Weissman A, Ravhon A, Horowitz E, Levran D (2010). Re: Cryopreserved-thawed human embryo transfer: Spontaneous natural cycle is superior to human chorionic gonadotropin-induced natural cycle. Fertil Steril.

[B19] Litwicka K, Mencacci C, Arrivi C, Varricchio MT, Caragia A, Minasi MG, et al (2018). HCG administration after endogenous LH rise negatively influences pregnancy rate in modified natural cycle for frozen-thawed euploid blastocyst transfer: A pilot study. J Assist Reprod Genet.

[B20] Mizrachi Y, Horowitz E, Ganer Herman H, Farhi J, Raziel A, Weissman A (2021). Should women receive luteal support following natural cycle frozen embryo transfer? A systematic review and meta-analysis. Hum Reprod Update.

[B21] Montagut M, Santos-Ribeiro S, De Vos M, Polyzos NP, Drakopoulos P, Mackens S, et al (2016). Frozen-thawed embryo transfers in natural cycles with spontaneous or induced ovulation: The search for the best protocol continues. Hum Reprod.

[B22] Groenewoud ER, Cohlen BJ, Macklon NS (2018). Programming the endometrium for deferred transfer of cryopreserved embryos: Hormone replacement versus modified natural cycles. Fertil Steril.

[B23] Reichman DE, Stewart CR, Rosenwaks Z (2020). Natural frozen embryo transfer with hCG booster leads to improved cycle outcomes: A retrospective cohort study. J Assist Reprod Genet.

[B24] Chi Yan Lee V, Hang Wun Li R, Shu Biu Yeung W, Pak Chung HO, Yu Ng EH (2017). A randomized double-blinded controlled trial of hCG as luteal phase support in natural cycle frozen embryo transfer. Hum Reprod.

[B25] Lee Y-J, Kim Ch-H, Kim D-Y, Ahn J-W, Kim S-H, Chae H-D, et al (2018). Human chorionic gonadotropin-administered natural cycle versus spontaneous ovulatory cycle in patients undergoing two pronuclear zygote frozen-thawed embryo transfer. Obstet Gynecol Sci.

[B26] Weiss A, Baram S, Geslevich Y, Goldman S, Nothman S, Beck-Fruchter R (2021). Should the modified natural cycle protocol for frozen embryo transfer be modified? A prospective case series proof of concept study. Eur J Obstet Gynecol Reprod Biol.

